# Mechanical and Thermo-Mechanical Performance of Natural Fiber-Based Single-Ply and 2-Ply Woven Prepregs

**DOI:** 10.3390/polym15040994

**Published:** 2023-02-16

**Authors:** Hafsa Jamshaid, Rajesh Kumar Mishra, Vijay Chandan, Shabnam Nazari, Muhammad Shoaib, Laurent Bizet, Tatiana Alexiou Ivanova, Miroslav Muller, Petr Valasek

**Affiliations:** 1 School of Engineering and Technology, National Textile University, Faisalabad 37610, Pakistan; 2 Department of Material Science and Manufacturing Technology, Faculty of Engineering, Czech University of Life Sciences Prague, Kamycka 129, 165 00 Prague, Czech Republic; 3 Department of Sustainable Technologies, Faculty of Tropical Agriscience, Czech University of Life Sciences Prague, Kamycka 129, 165 00 Prague, Czech Republic; 4 Normandie Université, UNIHAVRE, LOMC, CNRS UMR 6294, 76600 Le Havre, France

**Keywords:** prepregs, weaving, thermoplastic, tensile properties, dynamic mechanical analysis, thermal properties, thermos-gravimetric analysis, differential scanning calorimetry

## Abstract

This paper presents a study conducted on prepregs manufactured by a novel method for the impregnation of a thermoplastic matrix. Different composite prepregs based on polypropylene and reinforced with natural fibers (e.g., basalt and jute fibers) were developed. The mechanical and dynamic mechanical properties were investigated. DMA tests were conducted at 1 Hz frequency and properties such as storage modulus and damping (tan δ) were evaluated. The overall mechanical properties of the basalt fiber composites were found to be superior to that of the jute fiber-based samples. Thermo-gravimetric analysis (TG/DTG) of the composite samples showed that the thermal degradation temperatures of the basalt-based composites shifted to higher temperature regions compared to the PP or jute fiber composites. The addition of basalt fiber considerably improved the thermal stability of the composite samples. Microscopic images of the tensile fractured composite samples illustrated better fiber–matrix interfacial interaction due to the novel technology of prepregs. Single-ply and 2-ply prepregs showed significantly superior mechanical, thermal, and thermo-dynamical performance compared to the control sample (pure PP). 2-Ply composites demonstrated higher modulus, tensile strength, and storage modulus due to the higher fiber volume fraction. Basalt-based samples showed a minimum weight loss of about 57% up to 700 °C in contrast to 96.05% weight loss in the jute-based samples and 98.4% in the case of pure PP. The heat resistance index (T_HRI_) is more than twice for basalt compared to jute and PP. Furthermore, the superior thermal stability of basalt is reflected in its DSC curves, showing the highest endothermic peak. The technique of using the resin in the form of thermoplastic yarns offers cost effective and efficient alternatives for composite manufacturing.

## 1. Introduction

The research and application of degradable materials have received more and more attention in recent times. Environmental issues are affecting the composite industry, where the issue of sustainability is even more significant. Petroleum-based materials cause severe pollution, and therefore, the global emphasis is on environmental protection and sustainable development. Typical fibers used as reinforcement in composites are glass or carbon fibers due to their superior mechanical properties, but they are not environmentally friendly [1]. Basalt is a fiber that is obtained from rocks and has a generic name for solidified lava, which comes out of volcanoes. It is found in almost every country across the globe, which makes it widely preferred as a sustainable alternative. When this melt is quickly quenched, it solidifies into a glass-like nearly amorphous solid and the production process does not require the addition of secondary chemical components, as in the case of glass fibers [2]. Basalt fiber exhibits excellent mechanical performance, similar to those of glass fibers [3]. Its cost is lower than S-glass, making it suitable for the replacement of glass fibers in various industries. The higher thermal stability coupled with higher mechanical properties makes basalt fiber-based composites a better material that can sustain in harsh environments [4].

Natural fiber reinforced polymer composites are being widely used in aerospace and automotive applications. This is due to their low density and cost, better biodegradability, and higher specific strength and modulus, which has replaced E-glass fiber reinforced polymer composites [5]. In fiber reinforced composites, the use of renewable natural fibers such as ramie, jute, sisal, hemp, bamboo, oil palm fibers, banana, etc. as a reinforcement is increasing. Among these fibers, jute is of particular interest as the production of jute is excessive in Asian countries (Bangladesh and Eastern part of India) and composites made of jute fibers have moderate flexural and tensile properties compared with other natural fibers as studied by several researchers [6]. Its composites incur a lower processing cost and production energy, they are easily available, and exhibit moderate to high mechanical properties compared to several petroleum-based fibers. Jute fiber is utilized in hulls, surfboards, sports goods, swimming pool linings, construction panels, and vehicle bodywork [7].

The use of thermoplastic composites has significantly increased in recent years [8]. High fracture toughness, ease of recycling, fast production processes, weldability, and lower costs are the benefits of thermoplastic composite materials [9,10]. This means that the weight of composite parts may be reduced without sacrificing the structural stability or mechanical performance, and this can be achieved by using thermoplastic resins. Recent studies have shown that thermoplastic resins can be used for high performance fibers as well as natural fibers. Synthetic fibers (e.g., carbon, glass, polypropylene (PP)) and natural fibers like hemp, flax, jute, etc. are commonly used in composite materials for acoustic, mechanical, and thermal advantages [11,12,13]. Usually, the strength of a composite material depends on the type and content of the reinforcement. However, when the fiber content (volume fraction) is increased above 50%, there is deterioration in the mechanical performance due to insufficient wetting of the fibers by the matrix [14]. The effect of the fiber volume fraction on the mechanical, water absorption, and fire resistance properties of basalt and pineapple leaf fiber reinforced polyester composite panels has also been investigated [15,16].

Polypropylene (PP) is a significant market contributor because of its lower environmental impact through increased recyclability, ease of processing, and cost. PP composites have weatherability and chemical resistance, in addition to the cost aspect [17]. These composites are widely used in automotive, construction, and industrial applications. Different studies have reported the properties of composites using polypropylene resin, especially with basalt fiber as an alternative to glass fiber [18]. Jute and polypropylene (PP) braided textiles enhanced the mechanical characteristics of composites reinforced with jute and virgin polypropylene. Composites based on polypropylene are favored for car bumpers, front and side protection, and spoilers [19].

Natural fiber-reinforced polymer composites are being widely used in aerospace, marine, automotive, and health care applications due to their sustainability, low cost, and ecofriendly nature. These composites can be manufactured through conventional and advanced manufacturing techniques to avoid internal and external defects [20]. The optimization of process parameters of composite manufacturing techniques is essential for manufacturing high-quality components. However, studying many parameters through experimental approaches is time-consuming and labor-intensive. Modern technology has made it possible to monitor the complete manufacturing process “in situ” due to its high spatial resolution and very short acquisition time [21]. The reinforcing fibers can be bound in a variety of ways in a sheet form to handle them properly. The methods of binding the fibers together affect the properties of the final composites. Different techniques such as single-ply binding, weaving, knitting, braiding, etc. are used for this purpose. Two or more types of fibers of the reinforcing phase embedded in a matrix constitute a hybrid composite with better mechanical qualities in several instances. Single-ply (UD) composites have certain advantages over other methods [22].

Adhesion is one of the key considerations during composite manufacturing as it affects the load transfer between the fiber and the matrix and therefore the performance during their service life. In the case of thermoplastic composites, the interface region is crucial as the viscosity of the matrices is quite high, which inhibits the proper impregnation of the reinforcement. To overcome this problem, one solution is to reduce the flow distance of resin through the reinforcement. In order to overcome the limitation of flow distance, prepregs can be used. Prepregs are composite reinforcing materials that have been pre-impregnated with a thermoplastic or thermoset resin. While the production procedure is quicker for prefabricated parts, they are cured by hot compression molding. Therefore, it is beneficial to use thermoplastic prepregs over thermoset composites since they do not need a second curing step. Single-ply (UD) composites have been created by wrapping parallel sheets of yarn around a metal frame [23]. However, it has certain disadvantages such as a lot of waste produced especially for complicated parts, as variations in the positioning of reinforcement lead to a wide range of the final structures and the fiber volume percentage is not uniform. In high-end applications such as aircraft and automobiles, automated fiber placement (AFP) is chosen for production procedures. AFP reduces waste by reducing the number of joints in the component-part manufacturing process. It also increases the deposition speed, fiber alignment precision, automation, and repeatability while reducing the probability of variation. The disadvantage of this method is that when fibers shrink in various directions, residual stresses are created that might alter the form and dimensions of the composite [24]. To produce high-quality composites at a reasonable price, the most important consideration is cost-effective prepreg manufacturing. Researchers are investigating different method to produce cost effective prepregs. In the literature, knitted prepregs have been reported [24], but there is limited information about woven prepregs from thermoplastic components.

The aim of the current study is twofold: first to fabricate woven 2-ply thermoplastic composite prepregs by using environmentally friendly fibers and the other is a reduction in time by using hot compression molding of thermoplastic filament yarns. Polypropylene reinforced with jute and basalt fibers are made into woven structures. In these composites, the fabrication process is easier and efficient with a lower amount of waste. The novelty of this work is the simplified methodology to develop the thermoplastic prepregs (i.e., lamina (single-ply in the same orientation) and 2-ply (plies at different angles)). The mechanical and thermal properties of the single-ply and 2-ply preforms were studied. This is a low cost, more productive continuous process suitable for bulk production and can be easily transported without any issues of shelf life.

## 2. Materials and Methods

### 2.1. Materials

Jute (J) and polypropylene (PP) yarns were purchased from a local manufacturer in Faisalabad, Pakistan, while the basalt yarns were obtained from the company Kamenny Vek (KV) (North Carolina, USA). The linear density of all yarns was 300 tex.

Jute is a long, soft, shiny vegetable fiber that can be spun into coarse, strong threads. It is one of the most affordable natural fibers and is second only to cotton in the amount of global production. It falls into the bast fiber category and is 100% bio-degradable, recyclable, and thus environmentally friendly. It is a thermoset polymeric fiber. The idea of current research was to use these common polymeric fibers in the weft and warp to create woven structures and to further investigate the compatibility in composite manufacturing.

Polypropylene is a thermoplastic polymer and can be made by polymerizing propylene molecules. It is a by-product of oil refining processes and is a low-cost polymer with versatile applications. It has the lowest density among all of the synthetic fibers. PP possesses several useful properties such as high heat distortion temperature, transparency, flame resistance, dimensional stability, and high impact strength, which widen its application.

Basalt fiber is obtained from natural basalt, which is a dark colored, fine grained solidified volcanic rock. Basalt is a common term used for a variety of volcanic rocks. A hard, dense, inert rock found worldwide, basalt is an igneous rock (i.e., it melts when heated like thermoplastic materials), which is solidified volcanic lava. Basalt originates from volcanic magma and flood volcanoes, and is a very hot fluid or semi fluid material under the Earth’s crust that solidifies in the open air. Basalt flows cover about 70% of the Earth’s surface, in which SiO_2_ accounts for the main part, followed by Al_2_O_3_, then Fe_2_O_3_, FeO, CaO, and MgO. For this reason, basalt rocks are classified according to the SiO_2_ content as alkaline (up to 42% SiO_2_), mildly acidic (43 to 46% SiO_2_), and acidic basalts (over 46% SiO_2_). Only acidic type basalts satisfy the conditions for fiber preparation. High silica contents are required to obtain a glass network.

The details of the fibers and yarns are given in Table 1.

### 2.2. Methods

**Figure 1:**
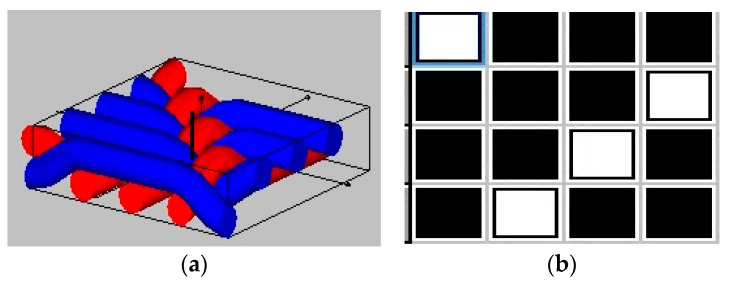
Weave structure: (**a**) 3D pattern, (**b**) graphical pattern.

All fabric samples were produced on the CCI loom with the same construction for all fabrics: 12 threads/cm in warp and 8 threads/cm in weft using a 1/3 twill weave. Basalt and jute yarns were used in the warp direction while polypropylene yarn was used in the weft direction. All of the fabric variants were measured, according to the standardized process. The weave pattern is shown in Figure 1.

The samples were developed with the parameter levels as given in Table 2.

Table 3 shows the list of samples and their codes.

#### 2.2.1. Manufacturing of the Composite Laminates

**Figure 2:**
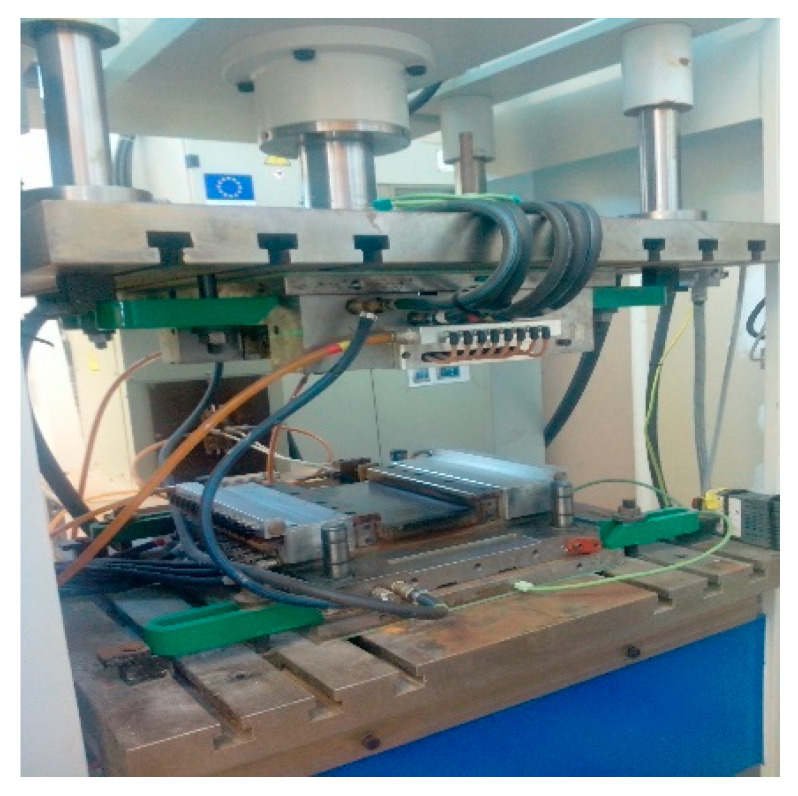
Compression molding device.

The induction-heated compression molding machine, as shown in Figure 2, was used to produce lightweight thermoplastic composite prepregs. The samples were placed in a compression molding machine by using a releasing agent (ZYVAX semi-permanent multiple releasing agent) on the plate. For impregnation, the composite samples were heated at 180 °C for 3 min for proper infusion of the matrix. A 40 bar pressure was applied to produce compact composites. Samples were weighed before and after impregnation to determine the fiber volume fraction (Vf). Single-ply composites had a Vf = 0.33, and the 2-ply samples had a Vf = 0.47.

#### 2.2.2. Testing

After the composite prepregs were prepared, the specimens were cut according to required size for mechanical, thermal, and thermo-mechanical testing.

#### 2.2.3. Static Mechanical Testing

For static mechanical testing, a TIRA 2300 (LaborTech s.r.o., Opava, Czech Republic) universal testing machine was used. The test was performed according to EN ISO 527-5. All tensile tests were performed at room temperature. For each property, 10 measurements were taken. The mean and standard deviation were calculated.

#### 2.2.4. Dynamic Mechanical Analysis

A dynamic mechanical analyzer, DMA 40XT RMI (Anton Parr, Prague, Czech Republic), was used to characterize the storage modulus (*E*’) and damping factor (tan δ). The samples were tested using three-point bending mode at a frequency of 1 Hz in temperature scan mode. The DMA test was executed from room temperature to 100 °C at a heating rate of 3 °C/min. This test was performed according to EN ISO 6721-1. For each sample, five measurements were taken.

#### 2.2.5. Thermo-Gravimetric Analysis

The Mettler Toledo TGA/SDTA851e instrument (Mettler Toledo, Prague, Czech Republic) was used to study the thermo-gravimetric behavior (thermal stability and degradation) of the composite prepregs. Thermo gravimetric analysis was performed under a dynamic nitrogen atmosphere. The samples were heated from 25 °C to 700 °C at a heating rate of 10 °C/min to yield the decomposition temperature, weight loss %, and maximum decomposition peak.

#### 2.2.6. Differential Scanning Calorimetry

Thermal analysis was carried out by a differential scanning calorimeter (DSC 6 Perkin Elmer instrument, Prague, Czech Republic) using Pyris^TM^ software-version 11 (Perkin Elmer, Berlin, Germany). The samples were treated with a heating rate of 10 °C/min from room temperature to 400 °C and then cooled in a nitrogen atmosphere with a flow rate of 20 mL/min.

#### 2.2.7. Microscopic Analysis/Fractography

The optical images of all of the tested samples were also obtained by using an optical microscope (OPTIKA C-B10, Optika S.r.l., Bergamo, Italy). Scanning electron microscopy (SEM) from (Tescan s.r.o., Brno, Czech Republic) was used to observe the cross-sectional images of the fractured samples. The samples for scanning electron microscopy were prepared with Quarum Q150R ES, which uses gold-plating with an argon gas atmosphere. The thickness of the gold plating was 2 nm using a sputter current of 20 mA.

The scanning electron microscope TESCAN MIRA 3 GME was used for this purpose. The samples were visualized in a nitrogen atmosphere with a SE (secondary electron) detector, with an acceleration voltage of 10 kV. The working distance was maintained at 16–32 mm in scan mode.

## 3. Results and Discussion

### 3.1. Tensile Properties

The tensile properties of a composite provide an indication of how it will respond to forces that are applied in tension. For the mechanical testing, a TIRA 2300 (LaborTech s.r.o., Opava, Czech Republic) universal testing machine was used following the EN ISO 527-5 standard. The results of the tensile testing are given in Table 4.

The tensile strength of the prepregs reinforced with jute or basalt was significantly higher than the control sample (pure PP). The 2-ply samples showed higher strength and modulus compared to the single-ply structures. This was attributed to a higher fiber volume fraction in the plied structures. The basalt-based samples exhibited a higher tensile modulus and tensile strength compared to jute-based samples. This is attributed to the superior tensile properties of basalt. The prepreg samples exhibited a greater superior tensile modulus compared to the pure PP sample due to strong bonding between the reinforcing fibers and the PP resin.

**Figure 3:**
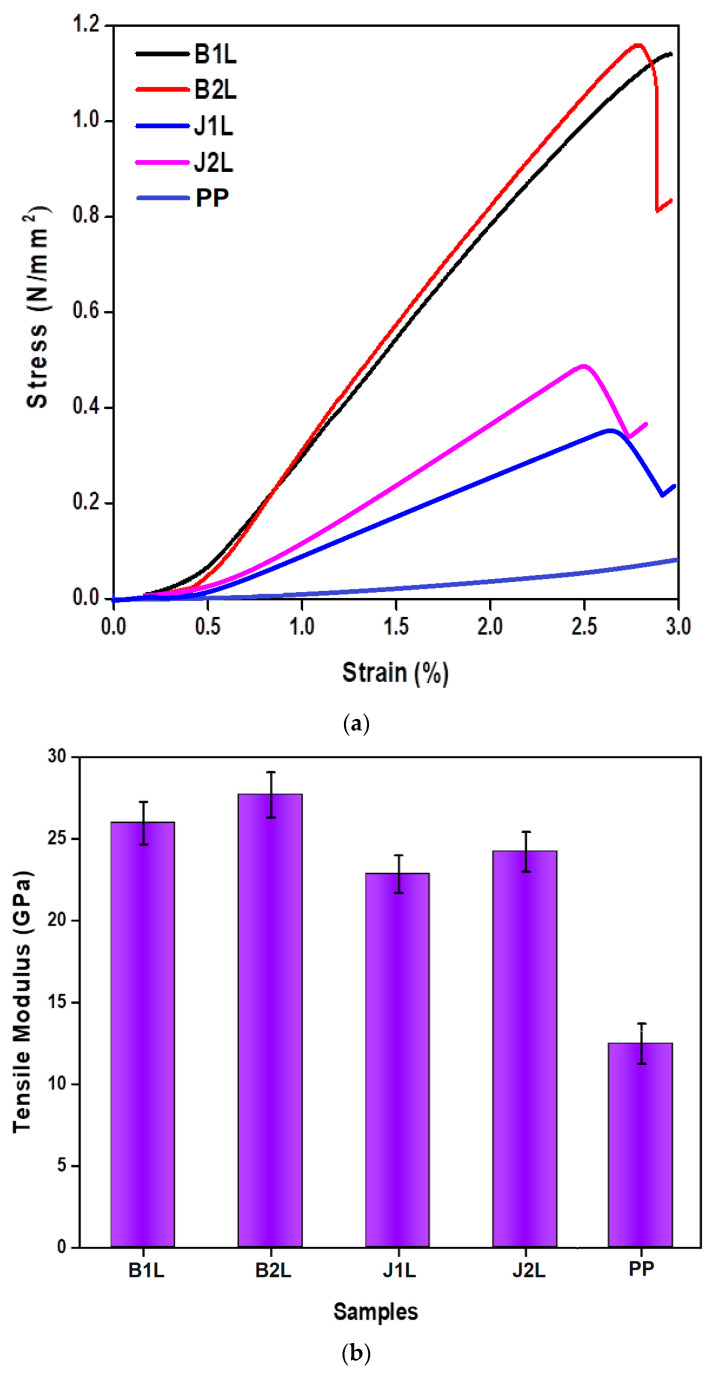
Tensile properties of the composites. (**a**) Stress–strain behavior of the composites vs. the control sample (PP). (**b**) Tensile modulus of the composites vs. the control sample (PP).

Figure 3a shows the stress–strain behavior of all the different composite prepregs alongside the control sample (pure PP). Composite sample B2L withstood the maximum stress due to the double layer of basalt in the weave structure bonded with the matrix. The basalt fibers had a high volume-mass fraction and exhibited a relatively higher tensile strength themselves [25,26,27,28]. While the B1L composite sample showed comparable results with B2L, it resulted in a higher elongation to break as it was composed of only one layer of basalt fabric. The B1L composite bore a lower level of stress due to the lower fiber content and the one-dimensional weave structure bonded with the matrix. The composite samples J1L and J2L withstood much lower stress than the basalt-reinforced composites as the jute fiber itself has a relatively lower tensile strength compared to basalt. Among the jute-based composite samples, the J2L structure composite showed better results with respect to the stress–strain behavior as the number of layers (combined effect) made the composite effective to the stress/load bearing capacity. The elongation at break for the single-ply basalt-based sample was higher than the 2-ply samples. The double-layered sample had a higher fiber volume fraction and thus resisted the deformation to a higher extent. A similar trend was also observed between the J1L and J2L samples. Jute, being a natural origin fiber, inherently shows irregularities in the fiber cross-section and thus the fabric thickness. In the composite sample composed of jute, there were a greater number of weaker links between the layers of fabric and a higher variation was observed. In general, the elongation to break for the basalt and jute reinforced composite prepregs was substantially lower than the control sample (pure PP).

Figure 3b shows the tensile modulus of the composite prepregs. From the figure, it can be clearly seen that the basalt–PP composite prepregs with two layers showed better results compared to the single layer of basalt in the weave structure. This is due to the compactness of the multilayered samples, which resulted in higher stiffness. A similar trend was observed in the case of the jute–PP composite prepregs. The tensile modulus increased almost twofold compared to the control sample (pure PP resin).

#### Fractography

**Figure 4:**
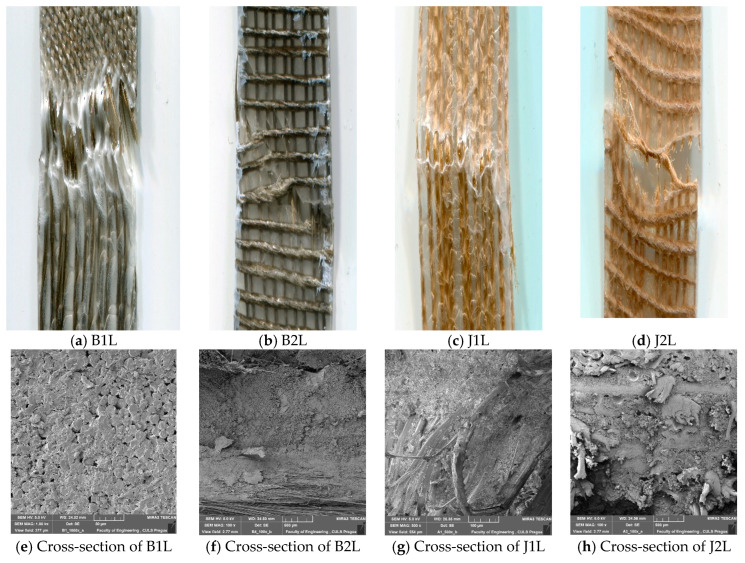
(**a**–**d**) Optical images of the fractured samples and (**e**–**h**) the SEM images of the cross-section.

The fiber–matrix interface is critical to determine the performance of the composites. In order to see the fiber–matrix interface, optical images were taken. Figure 4 shows the images of the cracked specimens. Images (a–d) show the longitudinal view by optical microscopy. Images (e–h) show the cross-sectional views taken with the scanning electron microscope (SEM).

It was observed that tensile failure occurred in a direction perpendicular to the loading. Cracks initiated perpendicular to the loading direction by separation between the yarn and the matrix (i.e., fiber/matrix debonding at the interface). On the application of further force, it resulted in the detachment of fibers from the matrix. The images showed a sharp rupture and no visible voids. The damage mechanism was similar for all the composite prepregs with basalt as well as jute reinforcement. In the 2-ply samples, the crack propagation was slowed down by increasing the fiber volume fraction perpendicular to the load. As the fiber content was higher in this case, the increase in interfacial bonds between the matrix and fiber resisted the applied load effectively. Delamination was reduced compared to the single-ply composites, which shows the ability of the material to absorb stress with minimum damage.

### 3.2. Dynamic Mechanical Analysis

To determine the mechanical properties of the composite materials under dynamic thermal stress, DMA is required, which is particularly important in the research on the viscoelastic behavior of polymeric materials. Sinusoidal thermal and bending stresses are applied to the material, and the strain in the material is monitored. Storage modulus and damping factor are the important features of the composites that relate to stiffness and energy dissipation, respectively. The viscoelastic storage modulus is a property of composite materials that indicates their stiffness and reflects the energy stored in a sinusoidally strained sample. It demonstrates the influence of temperature on the storage modulus of composite samples and demonstrates how the storage modulus vs. temperature curve may be used to determine the stiffness, degree of cross-linking, and fiber/matrix interfacial bonding of the material under consideration [29,30]. The storage modulus indicates the degree of stiffness in a material and denotes the energy stored in the sample’s elastic structure. Storage moduli affect the mechanical properties of the composites, which means an improvement in the bending modulus also leads to an improvement in the tensile properties. The loss modulus denotes the viscous component of the sample, or the quantity of energy wasted, whereas the tan δ denotes the ratio of the loss modulus to the storage modulus and shows the energy dissipation of the whole composite structure. Results of the storage modulus between a temperature range of 30–100 °C are given in Table 5.

The storage modulus for all the samples gradually decreased with an increase in the temperature from 30 to 100 °C. Additionally, a decreasing trend was observed for tan δ in all of the samples including the control sample (pure PP).

**Figure 5:**
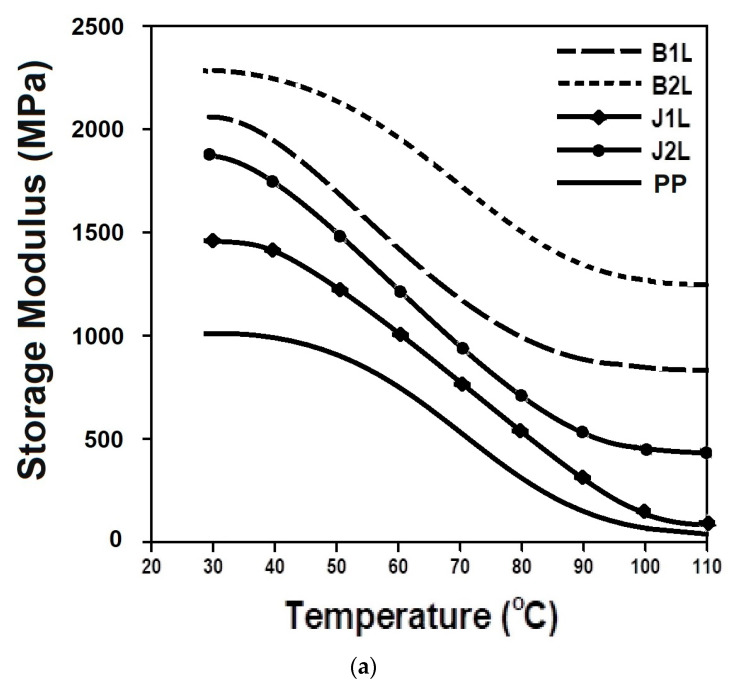

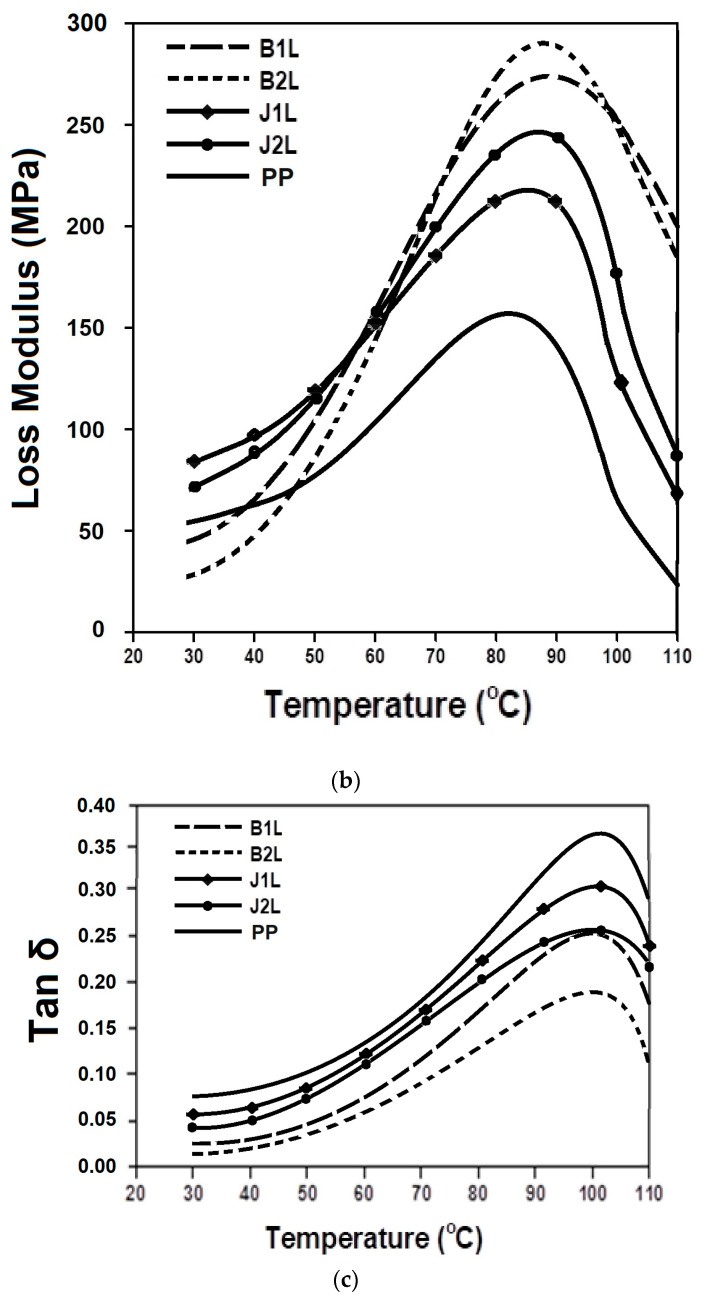
DMA analysis of the composites, (**a**) storage modulus, (**b**) loss modulus, and (**c**) tan δ of the samples.

The results of DMA are compared in Figure 5a in terms of the storage modulus, (b) loss modulus, and (c) tan δ.

It can be seen from Figure 5a that there was a decreasing trend in the storage modulus (decrease in stiffness) over the temperature range of 30–100 °C. Thermoplastic polymers are semi-crystalline and have a higher degree of orientation. At lower temperatures, molecules remain stiff due to their inability to resonate when subjected to vibrating loads, while at higher temperatures, the mobility increases, and the storage modulus drops off. Basalt-based composites were affected to a lower extent by the rise in temperature due to the inorganic nature of the fiber, which exhibited a stable nature over a wider range of temperatures. The B2L composite sample showed a higher storage modulus than B1L because the increase in the stiffness of the structure due to the presence of a higher volume fraction of basalt fibers allow for higher stress bearing. As the fiber content increases in the matrix, it leads to greater stress transfer at the interface. Due to uniform stress transfer from the fiber to the matrix, the sample results in higher dynamic mechanical properties. The trends of the curves obtained for the storage modulus and tensile modulus were similar. The jute-based samples showed a lower storage modulus compared to the basalt-based composites as the fibers themselves were less resistant to thermal stress. The effect of increasing the fiber volume fraction is similar to that of the basalt-based samples except for a small fluctuation that may be attributed to the irregularities and non-homogeneities of the jute fibers.

Figure 5b shows the loss modulus of the samples. The loss modulus indicates the amount of energy dissipated during the DMA test. It was observed that the loss modulus increased with an increase in temperature up to 80–90 °C, and after that, it started to decrease. As the temperature increased, there was a relaxation of the molecules in the polymers, which increased the polymer structural mobility. The basalt-fiber based samples showed a higher peak of loss modulus than the jute-based samples. Furthermore, the 2-ply samples showed a higher loss modulus than the single-ply composite prepregs. All of the composite samples exhibited a higher loss modulus peak compared to the control sample (pure PP). The result indicates that the addition of basalt and jute fiber as well as the increase in the fiber volume fraction considerably increased the effective stress transfer from the resin to the fibers.

It was observed in Figure 5c that the basalt fiber-based composite prepregs showed a lower tan (δ) compared to the jute-based samples. Furthermore, the 2-ply structures offered a lower damping factor compared to the corresponding single-ply composite prepregs. It could also be observed that there was an increasing trend in the loss tangent over the temperature range of 30–100 °C. This can be justified by the restriction of the motion of polymeric chains and the molecules resulting from the incorporation of rigid mineral fibers, which led to lower damping characteristics. More heat was produced, and higher deformation could not be recovered on the removal of external force as the material showed higher tan (δ) values. The basalt fiber-based 2-ply composite prepregs showed the lowest tan (δ) values, so a better impact resistance could be expected. All the basalt- and jute fiber-reinforced composite prepregs showed a higher storage modulus and lower tan (δ) compared to the pure PP over the temperature range of 30–100 °C.

### 3.3. Thermal Properties

The thermal properties of the materials indicate the physical response of a material with the application of short-term or long-term heat, which can affect the mechanical, physical, and chemical properties or may lead to degradation. The thermal properties of the composite prepreg samples were investigated across the temperature range that they may be subjected to. This was carried out in order to analyze and understand their service life before they begin to show signs of wear and tear. Thermogravimetric/differential thermogravimetric (TG/DTG) analysis and differential scanning calorimetry (DSC) were performed to study the thermal properties of the composite samples developed.

#### 3.3.1. Thermo-Gravimetric/Differential Thermo-Gravimetric (TG/DTG) Analysis

**Figure 6:**
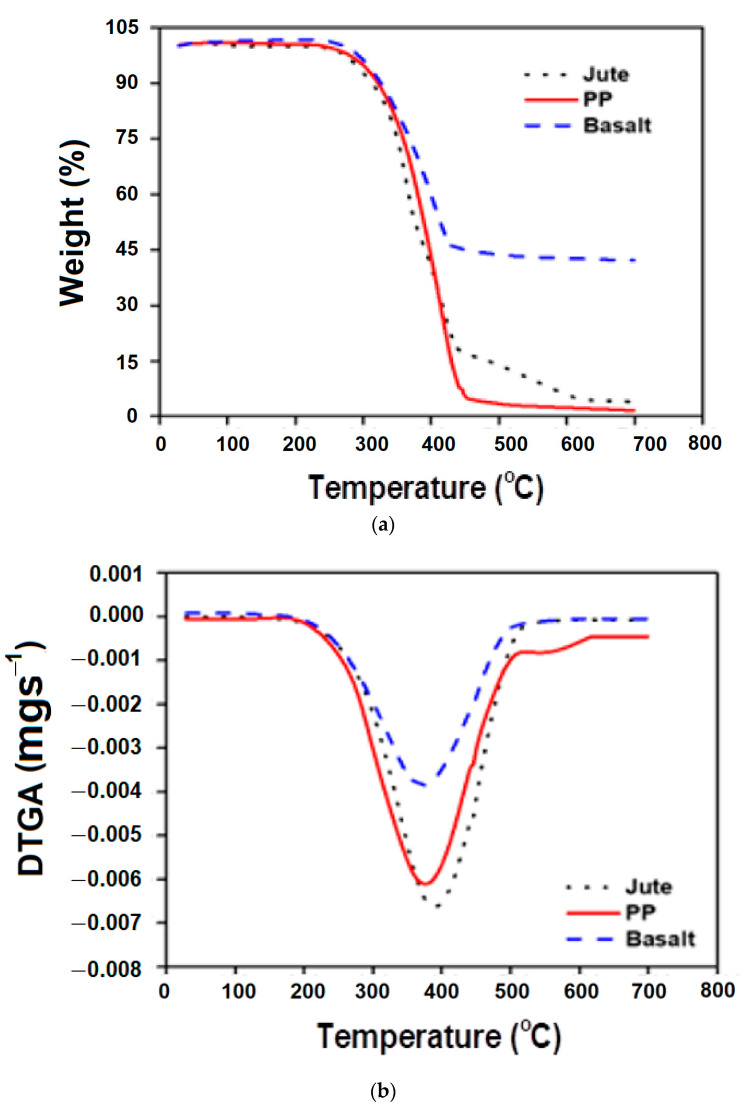
The thermal analysis of the composites: (**a**) TGA, (**b**) DTGA.

The TGA was used to determine the weight loss % of the composite prepreg samples as a function of increasing temperature. To assess the thermal stability of the composites, the TGA study was carried out for all composite samples with the basalt as well as jute fabrics. A comparative analysis of the various kinds of composite samples with respect to the pure PP sample is shown in Figure 6a. The derivative results of TGA were also plotted to obtain a clear picture of the events that occurred. The infection points in the TGA curve (higher weight loss %) were indicated by the peak on the DTG curve, which helps in obtaining the identification of events easier and the extraction of data.

The thermogram of the B/PP, J/PP, and pure PP showed a progressive weight loss as the temperature increased. The composite samples degraded in two phases: they became brittle and then they crumbled [31]. A progressive two stage thermal degradation occurred: the first stage is the degradation of the matrix, and the second stage is the degradation of fibers. At temperatures between 300 and 400 °C, the weight loss % was significant, which is the deterioration phase in polypropylene composites. The DTGA curves presented in Figure 6b also clearly indicate the existence of only one main mass-loss region, always located between 300 and 400 °C. This region can be attributed to the thermal decomposition of the polypropylene matrix, which has lower thermal stability. Other studies have reported findings that are almost identical to the present research [22,32,33]. PP has branched structures and alternative carbon atoms. The availability of reactive tertiary H atoms initiates its thermal degradation. Polypropylene is liable to chain degradation from exposure to heat. Oxidation usually occurs at the tertiary carbon atom present in every repeat unit. A free radical is formed, and then reacts further with oxygen, followed by chain scission to yield aldehydes and carboxylic acids. The melting temperature of PP is around 150–160 °C, which was observed by the peak in the differential scanning calorimetry (DSC).

The degradation temperature for natural fiber-based composites is often found between the decomposition temperature of the reinforcement and the decomposition temperature of the polymer matrix. As the matrix melts, the fibers dissolve more quickly. The temperature ranged from 410 to 485 °C in the second stage, which corresponds to the decomposition of fibers. When compared on the basis of thermal decomposition, the basalt and jute composites exhibited different levels of weight loss. This degradation behavior of the reinforcing yarns can be attributed to the variation in their chemical composition or content.

Because of the excellent thermal stability of the basalt fiber, it can be observed that the composite that was comprised of basalt fiber suffered a lower weight loss % compared to jute fiber when subjected to the temperature rise. After degradation of the PP matrix, a reduction in the weight of the samples occurred at around 450 °C due to the decomposition of the residual carbonate mineral in the fiber. After this, the weight loss still continued but at a much slower rate. At 700 °C, the total weight loss of the B/PP composites was about 57%. That is,, the total weight loss of the sample was much lower in the process of being heated from room temperature to 700 °C.

The jute fiber-reinforced composites had a higher weight loss % because of the lower decomposition temperature of jute. The thermal degradation in the jute resulted in char formation rather than molten drip. The formation of char residues may involve the initial physical desorption of water, intra molecular dehydration, formation of carboxyl and carbon–carbon double bonds, cleavage of glycosidic linkage, and the rupture of C–O and C–C bonds. This results in the condensation and aromatization of carbon atoms from each original pyranose ring to form discrete graphite layers [34]. Weight loss % for PP at 700 °C was 98.4%, 96.05% for the jute-reinforced sample, and 57% for basalt. 

The thermogravimetric analysis showed that there was no significant weight reduction observed for any sample up to 280 °C. Therefore, theses composites can withstand up to that temperature without compromising the final product quality.

#### 3.3.2. Calculation of the Heat-Resistance Index (T_HRI_)

The heat-resistance index (T_HRI_) is calculated using Equation (1) [34].

*T_HRI_* = *0.49* × [*T_5_* + *0.6* × (*T_30_* − *T_5_*)] (1)

where *T_5_* and *T_30_* correspond to the decomposition temperature of 5% and 30% weight loss, respectively, which were determined from Figure 6a. Since basalt is highly stable within the temperature range, it was heated until a 30% weight loss was observed at 1425.24 °C. Table 6 shows the TGA characteristic data and the values of the heat-resistance index (T_HRI_).

The results indicate that basalt is highly stable in the temperature range and shows a much higher heat resistance index. The jute fiber also showed a relatively higher T_HRI_ compared to PP. These observations are responsible for a higher thermal stability and lower mass degradation of the composite prepregs compared to the control sample (pure PP).

#### 3.3.3. Differential Scanning Calorimetry (DSC) Analysis

**Figure 7:**
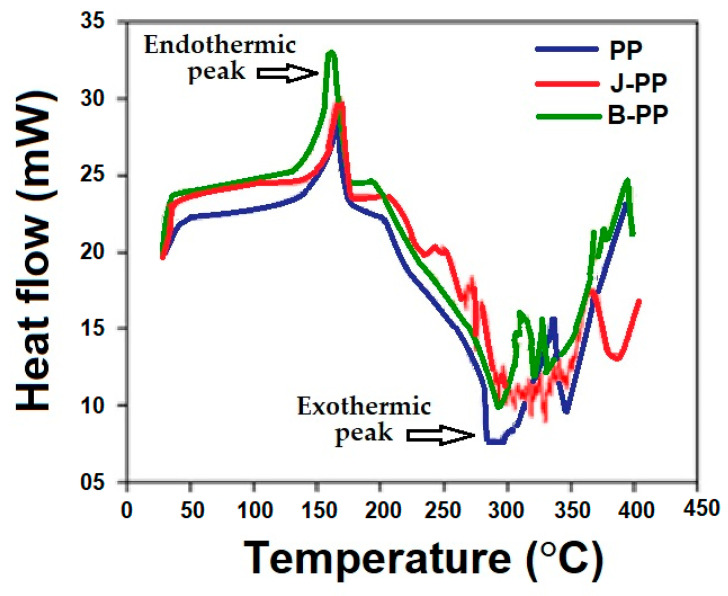
The DSC analysis of the composites.

Differential scanning calorimetry (DSC) monitors the temperature and heat flow resulting from different transitions as a function of time and temperature. This approach provides both qualitative and quantitative information on the physical and chemical changes that occur because of the endothermic and exothermic processes, respectively. The exothermic and endothermic peaks and magnitudes reflect the thermal phase change of the composites. The glass transition temperature (Tg) values of the matrices and the composites are critical characteristics that determine how the materials behave at various temperatures. Below this temperature, the materials become stiffer, and only a little deformation occurs when the materials are subjected to loading. When the temperature is raised beyond this point, the material shows rubber-like properties [35]. Figure 7 shows the DSC curves and the endothermic and exothermic peaks of the composites that were studied.

It can be observed that the endothermic peak emerged in the composite prepregs as the temperature was increased from room temperature to 150–160 °C. This peak was linked to the dehydration process of the composite samples and the matrix deformation started at this temperature. The melting temperature of PP is around 150–160 °C. The exothermic peaks in the developed composite samples were seen at around 280–300 °C, which was caused by the degradation of the matrix. Basalt offered higher peaks of heat flow, which indicates a better thermal stability of such composite materials. In the basalt–PP composite, the exothermic peak was different compared to jute–PP because of its higher thermal stability over jute. In both cases of basalt- and jute fiber/fabric-reinforcement, the exothermic peak was visible up until 300 °C, which indicates that the thermal stability of the reinforced composite was higher than that of the pure PP sample (control).

## 4. Conclusions

Basalt fiber, due to its environment friendliness, has evolved as a potential replacement for glass fiber. Composite prepreg samples were fabricated out of single-ply/2-ply basalt and jute reinforcements and their mechanical and thermal properties were investigated. The results reveal that basalt-fiber composites have superior mechanical and thermal properties compared to the jute-fiber-based samples. Storage modulus decreased with the increasing temperature due to the mobility of polymer chains for all of the composite samples. Two-ply structures offer superior thermo-mechanical properties to those of single-ply composites due to a higher fiber volume fraction. The thermal degradation of basalt-based composites was the lowest among all of the composites. The novel method of impregnating the fabrics by use of thermoplastic yarn (e.g., PP) can be used to replace conventional yarn winding techniques for single-ply/2-ply structures. Single-ply and 2-ply prepregs showed significantly superior mechanical, thermal, and thermo-dynamic performance compared to the control sample (pure PP). The basalt-based samples show a weight loss of about 57% up to 700 °C in contrast to a 96.05% weight loss in the jute-based samples and 98.4% in the case of pure PP. The heat resistance index (T_HRI_) was more than twice for basalt compared to jute and PP. Furthermore, the superior thermal stability of basalt was reflected in its DSC curves, showing the highest endothermic peak.

The proposed method uses the resin component in the form of thermoplastic fibers/yarns. This method can be used for any type of reinforcement, which is compatible to a thermoplastic resin. Additionally, it can be used for natural fibers and synthetic fibers. The composite made by the proposed method is only suitable for thin laminates as the melting of the thermoplastic component is the prime concern during hot compression molding. The properties of the composites prepared by this method are on par with composites of similar composition and dimensions. However, it cannot be used for thicker and bulkier composites, which can otherwise be better prepared with conventional impregnation techniques.

The method proposed here for thin laminates (single- and 2-ply prepregs) is quite simple, is easier, faster, and more cost effective compared to conventional methods such as resin transfer molding, injection molding, etc. This method can be used to assemble the reinforcement in different layers and directions for several planar applications. This concept seems to have promising applications and can be explored further.

## Figures and Tables

**Table 1:** The properties of the fibers and yarns used.

Properties	Basalt	Polypropylene	Jute
Diameter of fibers (micron)	12 ± 0.01	34 ± 0.01	18 ± 0.08
No. of filaments	890	300	--------
Linear density of yarn (tex)	300 ± 1.14	292 ± 1.02	296 ± 2.24
TPM (twists/m)	20 ± 1.00	30 ± 1.00	180 ± 1.00
Tensile strength (N)	92.75 ± 4.42	88.91 ± 3.07	41.43 ± 2.11
Tensile elongation (%)	1.29 ± 0.01	12.55 ± 0.72	1.39 ± 0.01
Tenacity (N/tex)	0.32 ± 0.01	0.23 ± 0.01	0.14 ± 0.01
Initial modulus (MPa)	9,378 ± 10.45	721 ± 7.42	3,741 ± 14.12

**Table 2:** The factors and their levels.

Factors	Level 1	Level 2
Reinforcing yarn	Basalt yarn	Jute yarn
Thermoplastic/matrix yarn	Polypropylene (PP)	Polypropylene (PP)
No. of layers/stacking sequence (angle in degrees)	1 (0)	2 (0/45)

**Table 3:** The samples developed.

Sample Code	Pattern of Layering
PP	Control sample (pure PP)
B1L	Basalt one layer
B2L	Basalt two layer
J1L	Jute one layer
J2L	Jute two layer

**Table 4:** The results of the tensile testing.

Sample Code	Maximum Tensile Stress (N/mm^2^)	Tensile Modulus (GPa)	Elongation to Break (%)
PP (control sample)	0.11 ± 0.01	12.25 ± 0.07	12.46 ± 0.41
B1L	1.15 ± 0.05	26.33 ± 1.12	3.56 ± 0.11
B2L	1.18 ± 0.04	27.75 ± 1.22	2.76 ± 0.09
J1L	0.31 ± 0.01	22.58 ± 1.08	2.68 ± 0.10
J2L	0.47 ± 0.02	23.67 ± 1.11	2.48 ± 0.18

**Table 5:** The results of the DMA testing.

Sample Code	Storage Modulus (MPa) at 30 °C	Storage Modulus (MPa) at 100 °C	Tan δ at 30 °C	Tan δ at 100 °C
PP (control sample)	998.42 ± 18.47	71.45 ± 1.21	0.07 ± 0.01	0.37 ± 0.01
B1L	2154.74 ± 24.42	1086.17 ± 22.72	0.02 ± 0.01	0.22 ± 0.01
B2L	2312.17 ± 27.16	1403.44 ± 24.08	0.01 ± 0.01	0.16 ± 0.01
J1L	1405.46 ± 28.33	114.24 ± 7.01	0.06 ± 0.01	0.29 ± 0.01
J2L	1803.83 ± 31.22	521.28 ± 10.86	0.04 ± 0.01	0.24 ± 0.01

**Table 6:** Calculation of the heat-resistance index (T_HRI_).

Sample	Weight Loss Temperature (°C)		Heat-Resistance Index (T_HRI_)
	*T_5_*	*T_30_*	
PP	301.15	411.21	179.92
Jute	303.22	421.44	183.33
Basalt	321.45	1425.24	482.02

## Data Availability

The data presented in this study are available in the article.
